# DFT-Based Permutationally Invariant Polynomial Potentials Capture the Twists and Turns of C_14_H_30_

**DOI:** 10.1021/acs.jctc.4c00932

**Published:** 2024-10-21

**Authors:** Chen Qu, Paul L. Houston, Thomas Allison, Barry I. Schneider, Joel M. Bowman

**Affiliations:** † Independent Researcher, Toronto, Ontario M9B0E3, Canada; ‡ Department of Chemistry and Chemical Biology, Cornell University, Ithaca, New York 14853, United States; § Department of Chemistry and Biochemistry, Georgia Institute of Technology, Atlanta, Georgia 30332, United States; ∥ National Institute of Standards and Technology, 100 Bureau Drive, Gaithersburg, Maryland 20899, United States; ⊥ Department of Chemistry and Cherry L. Emerson Center for Scientific Computation, Emory University, Atlanta, Georgia 30322, United States

## Abstract

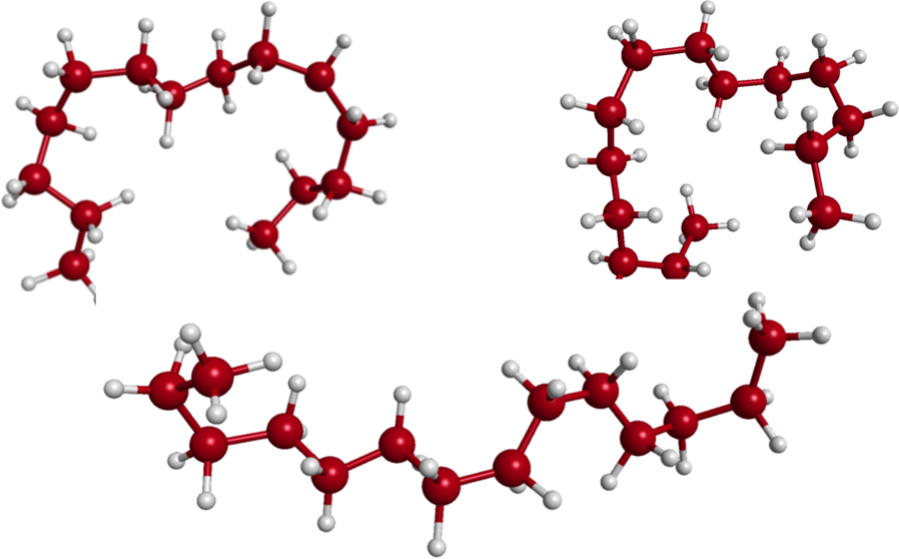

Hydrocarbons are ubiquitous as fuels, solvents, lubricants, and as the principal components of plastics and fibers, yet our ability to predict their dynamical properties is limited to force-field mechanics. Here, we report two machine-learned potential energy surfaces (PESs) for the linear 44-atom hydrocarbon C_14_H_30_ using an extensive data set of roughly 250,000 density functional theory (DFT) (B3LYP) energies for a large variety of configurations, obtained using MM3 direct-dynamics calculations at 500, 1000, and 2500 K. The surfaces, based on Permutationally Invariant Polynomials (PIPs) and using both a many-body expansion approach and a fragmented-basis approach, produce precise fits for energies and forces and also produce excellent out-of-sample agreement with direct DFT calculations for torsional and dihedral angle potentials. Going beyond precision, the PESs are used in molecular dynamics calculations that demonstrate the robustness of the PESs for a large range of conformations. The many-body PIPs PES, although more compute intensive than the fragmented-basis one, is directly transferable for other linear hydrocarbons.

## Introduction

Machine-learned potentials (MLPs) offer the promise of providing “first-principle” approaches for the full-span of molecular dynamics simulations.^1^ Among the panoply of machine-learned methods,^2^ permutationally invariant polynomials (PIPs)^3−5^ have been used for nearly 20 years to develop precise, high-dimensional potential energy surfaces (PESs) for molecules,^6−9^ clusters,^10−12^ many-body terms for water potentials,^13−16^ and atom-centered many-body representations for materials^17^ and molecular force fields.^18^ The precision of a PIPs PES for “rMD17 ethanol” was shown to be as good as the best performing ML methods and to be substantially faster (factors of 10 or more)^19^ than all the ML methods considered, i.e., GAP-SOAP,^20^ ANI,^21^ DPMD,^22^ sGDML,^23,24^ PhysNet,^25^ KREG,^26^ and pKREG.^27^ Similar factors in speed differences and high precision for PIPs were recently reported for 21-atom aspirin.^28^ In addition, PIPs have been used to fit more extensive and complex data sets than are given in the rMD17 data set^29^ for molecules with 10 or more atoms. Examples include formic acid dimer,^30^ glycine,^31^*N*-methylacetamide,^32^ acetyl acetone,^33^ and tropolone.^34^ PIPs have also been used effectively and extensively as inputs for Neural Network^35−37^ and Gaussian Process Regression^38^ for MLPs.

Given the high precision and speed of the PIPs approach, it is also important to “push” PIPs to even larger molecules, as well as more complex data sets. We report that here in the first of a series of papers devoted to alkanes C_*n*_H_2*n*+2_. In this paper, the focus is on C_14_H_30_. We present a large and complex data set of density functional theory (DFT)/B3LYP energies and gradients for this 44-atom hydrocarbon. Then we use our fragmented basis approach^39−41^ to fit this data set along with a novel, many-body PIPs approach that is inspired by previous MB PIPs methods.^15,42,43^ Although this paper focuses on the methods to generate the data set and the fits to it, we go beyond the usual precision metrics by presenting extensive results from molecular dynamics calculations, geometry optimizations and normal-mode analysis.

The remainder of the paper consists of a brief review of regression using PIPs. The fragmented-basis and the many-body PIPs approaches are then given. Computational details, beginning with the generation of the data set for training and testing are presented followed by numerical details of the two PIPs approaches. The results begin with the usual precision metrics, followed by an examination of numerous properties of the PESs, with a focus on chain flexibility. Timings for both PESs are detailed. This is followed by a discussion of the two PIP approaches and comments about related atom-centered ML methods. A short summary and conclusions completes the paper.

## Methods

### Permutationally Invariant Polynomial Bases

We start with a brief review of using a basis of permutationally invariant polynomials (PIPs) in linear regression for a machine-learned potential, *V*. The basic equation for this is given by
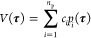
1where **τ** is the collection of Morse variables, *c*_*i*_ are linear coefficients, *p*_*i*_ are PIPs, and *n*_p_ is the total number of polynomials (and linear coefficients *c*_*i*_) for a given maximum polynomial order. The Morse variables are transformed internuclear distances *r*_*ij*_ between atoms *i* and *j*, i.e., *y*_*ij*_ = exp(−*r*_*ij*_/*a*).^3^ The range (hyper)parameter *a* is typically between 2 and 3 bohr; however, it can be optimized for special applications. A more revealing expression for a PIPs potential for a tetra-atomic molecule is given by
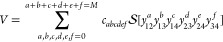
2where “” is the operator that symmetrizes monomials, and *M* is the maximum polynomial order. Our general software to perform this symmetrization and also to generate higher-order polynomials recursively has been described in detail.^4,5,44^ Also, a short video describing this software is available for the interested reader.^45^

This software has been applied to develop PESs for many molecules and complexes^6,7^ and most recently to the 21-atom aspirin molecule. To apply PIPs to even larger molecules, we proposed and tested a fragmentation scheme.^5,32^ However, the new method has not been applied to a molecule with more than 15 atoms. The remainder of this section is devoted to a description of the fragmented-basis approach to constructing PIPs and a new many-body method to construct a PIPs potential specifically for large molecules.

## Fragmented-Basis for PIPs Potentials

The formation (dissociation) of a given molecule from (into) its fragments is a ubiquitous general idea in computational chemistry; the overarching goal is to describe molecular properties from the union of its smaller fragments. In one approach, the total energy of molecule is given by the energy of fragments.^46,47^ This general idea has been extended to the construction of PIPs in order to obtain a PIPs basis describing the energy of the entire molecule without fragmenting the energy.^32,39^ A brief review is given below followed by details for application to C_14_H_30_.

### Fragmented Basis

To motivate the fragmented PIPs approach, recall that the PIPs basis can be generated by starting with a monomial,^4^ given by eq 3
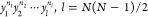
3where for simplicity the Morse variables are indexed by a single integer and where *N* is the number of atoms. For extended molecules many of the internuclear distances are large and so the corresponding Morse variables and corresponding monomials are nearly zero and can be pruned, i.e., discarded from the basis. This observation is the major motivation for the fragment-basis PIPs approach we proposed for large molecules.^32,39^

We do note that the straightforward way to prune the PIPs basis is to start with the full basis and then remove polynomials that are small. Of course a definition of “small” needs to be given and it must apply across the range of all Morse variables then compared to a threshold. Another approach, which is described in detail elsewhere,^5^ is to rank order the polynomials in magnitude over that range of Morse variables. Then the user can determine a cutoff based on desired size of the basis. However, this approach requires having the full PIPs basis, and this could be prohibitive for large molecules. To circumvent this problem, we proposed and tested a fragmented-basis method to obtain a PIPs basis without the full basis.^39^ This test was done for 12-atom *N*-methylacetamide, where a full PIPs basis was feasible and provided benchmark results. It was shown that the “conservative” choice of making the smallest number of fragments worked well. And there had to be substantial overlap of fragments. We will illustrate this in detail below for the present application. Then the final PIPs basis is just the union of these fragment bases. Finally, we stress that this approach is not the same as fragmenting the molecule and obtaining the energy as the union of energies of the fragments.

For C_14_H_30_ we considered a 3-fragment model for the basis. In this case the potential is given by
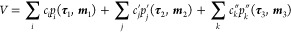
4where {*p*}, {*p*′}, and {*p*″} are PIPs bases for the *n*th fragment, *n* = 1,2,3, {*c*}, {*c*′}, {*c*″} are the corresponding linear coefficients, **τ**_*n*_ represent the set of corresponding Morse variables, and ***m***_*n*_ indicate a set of monomials built from the Morse variables for fragment *n*. By choosing the atoms in each fragment judiciously, one can keep those Morse variables for atoms that are close to one another while neglecting those for atoms that are far apart. It is important to have some overlap between the fragments, and permutational symmetry requires that Morse variables that permute in one fragment must similarly permute in any other fragments where they occur. Details about the fragments of C_14_H_30_ will be given in the Computational Details section.

### Many-Body Representations

A very different approach from the fragmented-basis is the many-body one. This approach has a long history in atomic physics, notably for Si and using models for these terms.^48,49^ A recent application to the noncovalent Ar atom 3-b interaction^50^ is also noteworthy. For noncovalent molecular applications, the “bodies” are molecules. A notable example is for water potentials, where the total energy of *N* H_2_O monomers is given by^15,42^
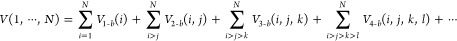
5where the *n*-body terms are described in detail below.

Next we present the many-body expansion using PIPs. There are two versions of this representation that we denote as “term-by-term” and “unified” here. We briefly review the “term-by-term” representation, which has been widely used, as we note below.

#### Term-by-Term Fitting

In the context of water potentials, *V*_1–*b*_ in eq 5 is the isolated monomer potential,^51^*V*_2–*b*_ is the intrinsic interaction between two water molecules, *V*_3–*b*_ is the intrinsic interaction between three water molecules, and *V*_4–*b*_ is the intrinsic interaction between four water molecules, etc. For the recent q-AQUA,^15^ q-AQUA-pol^52^ and MB-pol(2023)^16^ potentials, the 2-b, 3-b and 4-b terms are each separate PIPs fits to thousands of CCSD(T) 2-b, 3-b, and 4-b interaction energies, calculated separately for the dimer, trimer and tetramer.

This approach has also been used for heterogeneous systems, for example clathrate hydrates with different guest molecules such as H_2_,^10^ CH_4_,^53^ and CO_2_,^54^ and notably the hydrated proton.^55^

The many-body expansion for covalent interactions for a molecule is less common in the recent literature; however, there is a history using this approach for triatomic and tetraatomic molecules, as developed in the 1980s by Murrell and co-workers.^56^ A recent example of this approach, relevant to the present work on alkanes, was reported by Paesani and co-workers.^9^ In that work the “monomers” are CH_3_ and CH_2_ and the many-body series truncates at the 4-b terms, i.e., fragments with at most 4 carbon atoms. Each *n*-body term is fit using PIPs, as above. (This representation is augmented by long-range polarization terms, but this is not relevant to the present work.) The representation was applied to several hydrocarbons up to decamer, with encouraging results, albeit without extensive investigation of the final PES. This important paper was a “proof of principle” of the power of this approach. It was noted that the basic idea of using a many-body approach to fit a molecular potential had been suggested previously using fragmentation ideas.^47,57^

#### Unified Fitting

In the unified fitting approach, which we use here, the many-body form given by eq 5 is used; however, the full expression is used to fit the data in a single step, in contrast to the term-by-term fitting to the corresponding small cluster.

For hydrocarbons, and all two-element molecules, clusters, materials, etc., there are three types of 2-b interactions, which for hydrocarbons are CC, HH, and CH interactions. For 3-b and 4-b there are CCC, CCH, CHH, HHH and CCCC, CCCH, CCHH, CHHH, and HHHH interactions, respectively. We do not consider 5-b interactions here. These interactions are enumerated for all atoms in the molecule. For an *n*-body interaction among *N* atoms there are a total of  interactions. To limit the number of interactions, physical range cutoffs are used for each *n*-body interaction and we describe that here.

Proceeding, the potential up to 4-b interactions is given by
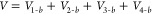
6where *V*_1–*b*_ is the total energy of 14 isolated carbon and 30 hydrogen atoms, which is a constant. The 2-b interaction is given by
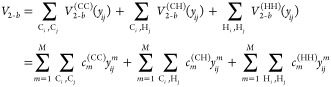
7where each 2-body term is just a linear expansion using a basis of powers of Morse variables for the corresponding CC, CH, and HH internuclear distances. Here we used the same total polynomial order *M* for simplicity, but they can be different for CC, CH, and HH. The actual values used in this work are presented in Computational Details. The 3-b term, *V*_3–*b*_ is given explicitly by the sum of 4 distinct 3-b interactions, namely
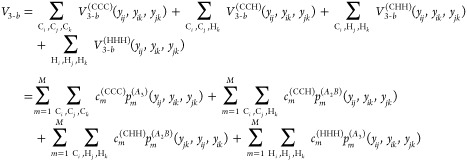
8where now the permutational symmetries of the two 3-b PIPs are *A*_3_ and *A*_2_B. Note that although CCC and HHH both use the same *A*_3_ PIPs, they use two different sets of coefficients; same for CCH and CHH. Finally, the 4-b term, *V*_4–*b*_, is given by five distinct terms, namely
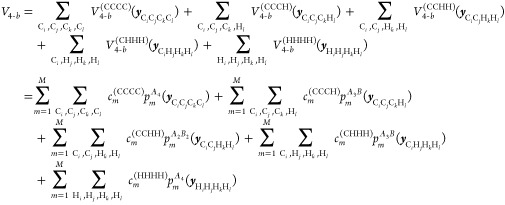
9

To summarize, the general permutational symmetry of the PIPs bases are *A*_4_, *A*_3_B, and *A*_2_B_2_. Also, we note that the distinct 2-b, 3-b and 4-b PIP bases can have different range parameters for the corresponding Morse variables, and different maximum polynomial orders. Once these bases are set up, the linear coefficients are {*V*_1–*b*_, ***c***^(CC)^, ***c***^(CH)^, ***c***^(HH)^, ***c***^(CCC)^, ***c***^(CCH)^, ***c***^(CHH)^, ***c***^(HHH)^, ***c***^(CCCC)^, ***c***^(CCCH)^, ***c***^(CCHH)^, ***c***^(CHHH)^, ***c***^(HHHH)^} from eqs 6–9, and they are optimized all at once in an overdetermined linear least-squares fit to the total energies of this molecule.

Details of the fragmented-basis and many-body approaches for C_14_H_30_, preceded by a description of the data set, are given in the next section.

## Computational Details

### Data Set

Creating a data set for subsequent fitting is a critical aspect of the goal to obtain a robust and precise PES. Many approaches are based on direct-dynamics, where configurations, energies and forces are obtained at a variety of temperatures (using NVT simulations) or total energies (using NVE simulations) via direct calls to software that generates these data. Our work using this approach dates back to 2003, where we used NVE direct(MP2)-dynamics to generate a data set that was fit using PIPs for CH_5_^+^.^58^ More recently, “MD17” data sets have been developed using NVT direct-dynamics at 500 K^29^ for a number of molecules. These data sets have been used extensively by the ML community to test methods. A recent critique of this data set noted the limited range of the configurations and energies obtained from this sampling^59^ and another more recent one noted the limitation to nonreactive potentials.^60^

In the current application, we continue to employ direct-dynamics, but given the size of the molecule and the need for long-time dynamics, DFT is not feasible. Instead, we use the well-known MM3 force field^61^ in NVT molecular dynamics at 100, 500, 1000, and 2500 K, using the Tinker package.^62^ Specifically, the “dynamic” program was used to calculate trajectories using the efficient and realistic MM3 force field,^61^ with an equilibration time of 100 ps followed by 1 ns (*T* = 500 K and *T* = 1000 K) or 2 ns (*T* = 2500 K) of simulation with a time step of 1 fs and a constant temperature Nosé–Hoover thermostat.

Several schemes to generate trajectories were used. In one, trajectories were calculated at each temperature starting from the MM3-optimized, linear configuration of *n*-C_14_H_30_. Configurations were collected every 100 fs, yielding a total of 40,000 configurations. At these configurations the energy and gradient were computed at the B3LYP/cc-pVDZ level of theory. Thus, only a small fraction, 1%, of the MM3 configurations were used to obtain the DFT energies and forces. The energies and forces were obtained using the Gaussian 16 computational chemistry package.^63^

In a second scheme, configurations from the 500 K direct-dynamics were obtained as follows. The head-to-tail distance (i.e., the C1–C14 distance) was calculated for all configurations from the simulation. A total of 15 configurations were selected from this set at 3.5, 4.0, 5.0, ···, 16.0, 17.1 Å. At each configuration, a single trajectory (1 ns) was computed at *T* = 100 K in the same manner as described above. The energy and gradient were calculated using the coordinates from the simulations at the B3LYP/cc-pVDZ level of theory for each configuration for a total of 150,000 configurations.

In the final scheme, 10,000 configurations from the *T* = 500 K simulation were optimized at the B3LYP/cc-pVDZ level of theory and vibrational frequencies were computed at the optimized geometry. Configurations were taken from each step in the optimization procedure if the energy was more than 0.25 kcal/mol above the energy of the fully optimized structure. The configuration of the optimized structure was used as well. In this manner, 82,529 additional configurations were generated at which B3LYP/cc-pVDZ calculations were done.

In total 272,532 configurations and corresponding B3LYP energies and gradients were obtained for training and testing of the model. (10,081 sets of vibrational frequencies at optimized structures are available; however, these were not used for training and testing the PIPs PESs. The vibrational frequencies of the optimized structure of linear C_14_H_30_ is included in this data set).

**Figure 1:**
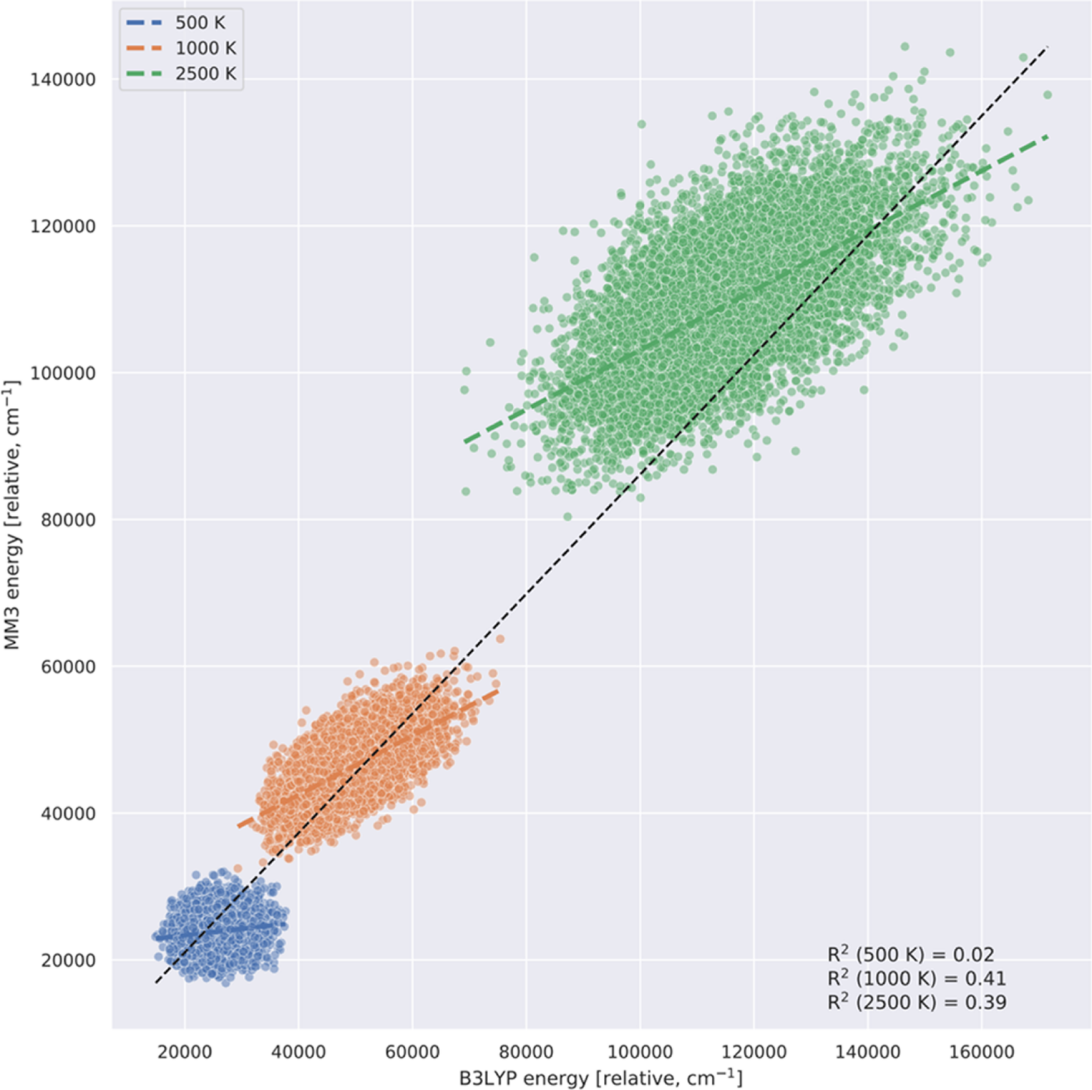
Correlation plots of MM3 and B3LYP energies at *T* = 500, 1000, and 2500 K. The dashed black line indicates a perfect correlation.

Before proceeding with details of training and testing, we examine the validity of the assumption that the MM3 force field provides a realistic data set of configurations to obtain DFT energies and gradients. This is addressed in Figure 1, where correlation plots of MM3 energies against B3LYP ones are given for data collected at 500, 1000, and 2500 K. As seen, there is qualitative correlation with the B3LYP energies, which span a large range from roughly 20,000 cm^–1^ to nearly 180,000 cm^–1^. This is a good indicator that the MM3 calculations do provide a diverse data set of configurations, which was the goal. Clearly though, there are large differences in the MM3 energies relative to the B3LYP ones, differences that provide strong motivation for going beyond the MM3 force field with the ML PESs we report here. Additional results showing DFT and MM3 energy histograms from the three temperatures and histograms correlated with the C1–C14 distance are given in the Supporting Information (SI).

### Selected Configurations from Training Data set

**Figure 2:**
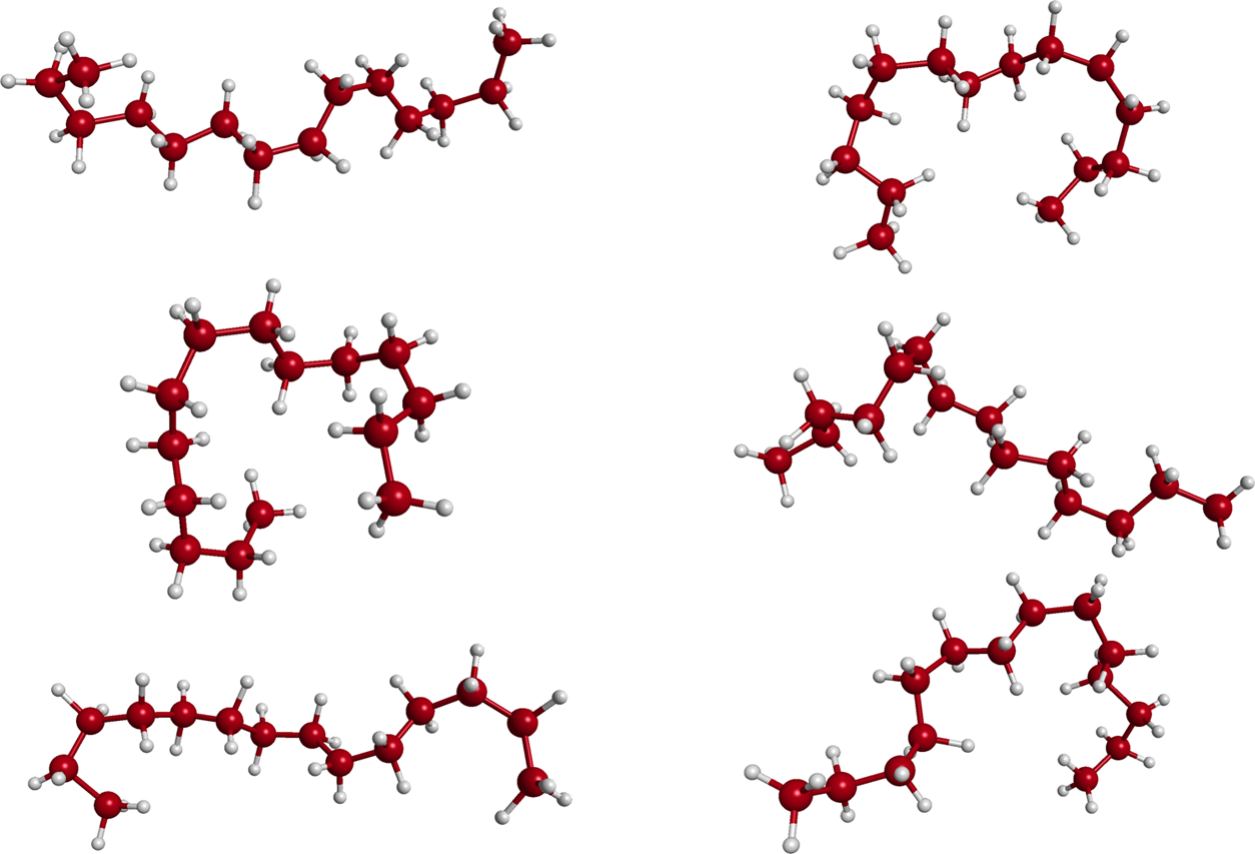
Selected configurations of C_14_H_30_ from the training data set.

The above procedure yielded a rich array of conformations of C_14_H_30_, a sample of which is shown in Figure 2. The massive flexibility of this alkane, shown in this figure, is not surprising given that the single bonds permit substantial dihedral motion. However, the energies of such configurations, while high, are well within the sampling at 1000 K. This is discussed in more detail below. The major point in showing these is to highlight the challenge a ML fitting method confronts to precisely describe this large diversity of conformations.

### Energy Distributions

**Figure 3:**
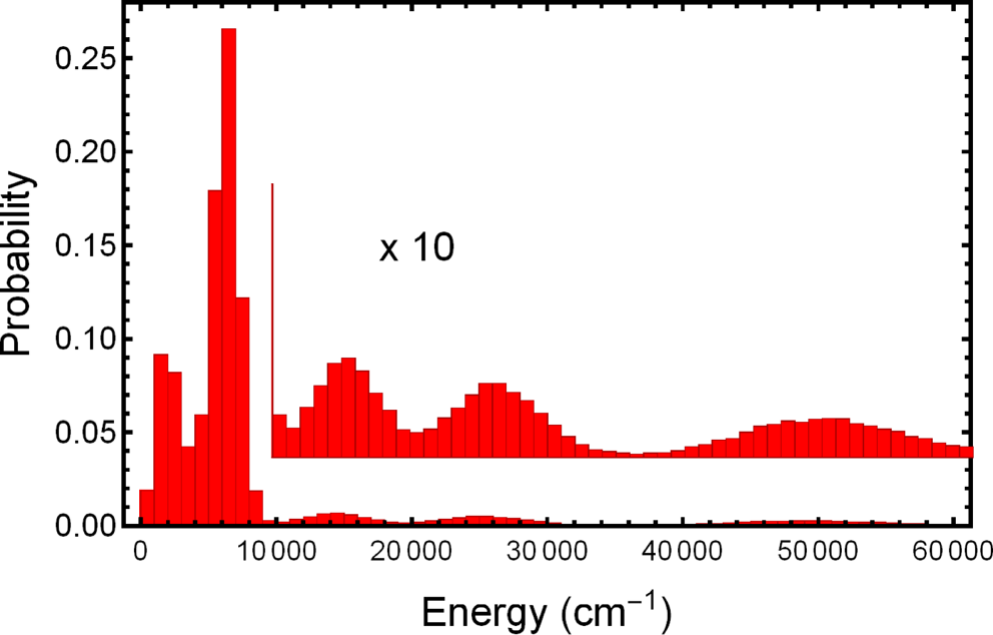
Energy distribution of the B3LYP data set used for fitting the PESs.

Histograms of MM3 and B3LYP energies, relative to their respective global minima, are shown in SI Figure SI-2 for 500, 1000, and 2500 K MM3 MD simulations. As seen, there is a large range of energies spanned by these (from roughly 0 to more than 150,000 cm^–1^). From this large data set, we selected a set up energies to 80,000 cm^–1^ (229 kcal/mol); and we stress that only the DFT/B3LYP energies are used in the PIPs fits. A histogram of the final data set of 253,646 energies, used in the fits, is shown in Figure 3. As seen, there is concentration of energies below 10,000 cm^–1^ and a roughly an equal number of energies spread broadly from 10,000 to 80,000 cm^–1^.

### Fragmented Basis and PES

To begin, we describe the procedure to determine the fragments used to form the PIPs basis. In order to obtain a robust PES, the fragmentation scheme must take account of the large range of configurations shown in Figure 2. A key aspect is the proximity of the two groups of three H atoms on each methyl rotor at each end of C_14_H_30_. So while the intermolecular Morse variables are very small for these two groups in the linear chain, they are not as small for many configurations. Thus, at least one fragment should include these six H atoms.

**Figure 4:**
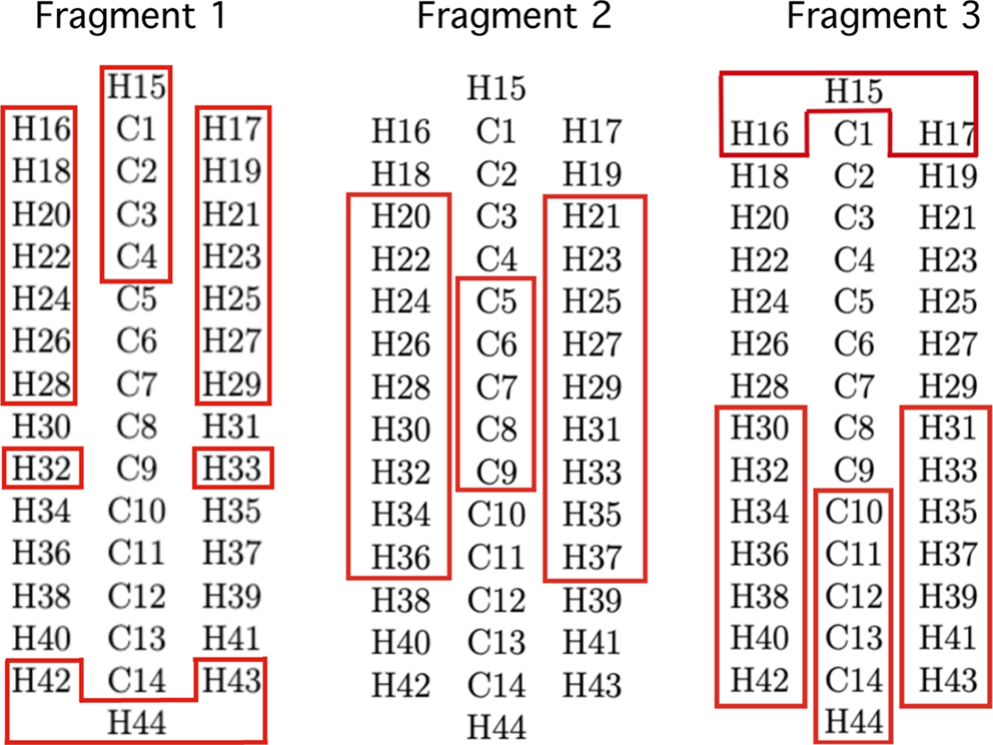
Schematic of the fragmentation scheme showing the atom labels and numbering.

The first task is to establish a numbering scheme for the atoms so that one can simply specify the atoms that will be in each fragment and compactly describe the permutational symmetry. For C_14_H_30_ a simple numbering system will suffice, as shown in Figure 4. Note that the specification of each atom has a type, here H or C, and a number indicating its position. It is important to stress that this linear arrangement of the carbon atoms in the figure is mainly for clarity. Although the global minimum (GM) does have this arrangement, it is clear from Figure 2 and the associated commentary that there are numerous nonlinear arrangements of these atoms.

The next step is to determine the desired permutational symmetry. Of course, we could insist that all carbons permute with one another and that all hydrogens permute with one another. Because this could be accomplished only with a single fragment, and because it would be nearly impossible to calculate such a basis, we turn to other possibilities.

The possibility that we will pursue is to have each of the three hydrogens on the end carbons permute with one another and each of the two hydrogens on the other carbons permute with one another. Note that the carbons would not permute with one another, but at normal energies this is unlikely to be physically relevant. The permutational symmetry for this scheme is denoted {3, 3, 2, 2, 2, 2, 2, 2, 2, 2, 2, 2, 2, 2, 1, 1, 1, 1, 1, 1, 1, 1, 1, 1, 1, 1, 1, 1}, where the “3s” refer to the hydrogens bonded to the end carbons, the “2s” refer to the hydrogens on the other carbons, and the “1s” refer to the carbons (which do not permute with one another in this scheme).

We next consider construction of the PES. The PES will be based on a fit to the data set, so if atoms never come close to one another in the data set, then the PES will never be able to calculate a proper energy for such close encounters. Thus, the next step in the process is to use the data set to evaluate which atoms come closest to one another. We accomplish this evaluation by calculating Morse variables for the geometries in the data set and recording, for all *N*(*N* – 1)/2 internuclear distances, the largest Morse variables, i.e., those corresponding to the shortest internuclear distances.

Consider now the logical choice of atoms in each fragment. Suppose that we want *n* fragments centered on *n* atoms whose identities are given by the numbers *p*_*n*_, *n* = 1, ···, *n*. Further, suppose that we want each fragment to have no more than *a*_*n*_ atoms. Finally, we need to account for the chosen permutational symmetry; that is, any fragment that includes a permuting atom must also include all the other atoms that permute with that atom. (If this rule is not obeyed, the overall basis will not carry the desired permutational symmetry.) To determine the best atoms for each fragment, we start with *p*_1_, include any atoms that permute with it, and then include other atoms (and those that permute with them) prioritizing those atoms that have the largest data set Morse values in combination with *p*_1_. We include all atoms such that the total number of atoms for this fragment is ≤*a*_1_. We move similarly through the other fragments, noting that there may be some desirable overlap of atoms between fragments.

For the permutational symmetry described above, we choose 3 fragments with no more than 24 atoms each and with *p*_1_ = 1, *p*_2_ = 7, and *p*_3_ = 13. The results of the procedure for choosing the best fragments are one fragment of 24, and two of 23 atoms. The first fragment has a permutational symmetry of {3, 3, 2, 2, 2, 2, 2, 2, 2, 1, 1, 1, 1} with atoms {15, 16, 17, 42, 43, 44, 18, 19, 20, 21, 22, 23, 24, 25, 26, 27, 28, 29, 32, 33, 1, 2, 3, 4} (in the permutational order). The second has a permutational symmetry of {2, 2, 2, 2, 2, 2, 2, 2, 2, 1, 1, 1, 1, 1} with atoms {20, 21, 22, 23, 24, 25, 26, 27, 28, 29, 30, 31, 32, 33, 34, 35, 36, 37, 5, 6, 7, 8, 9}, and the third has permutational symmetry of {3, 3, 2, 2, 2, 2, 2, 2, 1, 1, 1, 1, 1} with atoms {15, 16, 17, 42, 43, 44, 30, 31, 32, 33, 34, 35, 36, 37, 38, 39, 40, 41, 10, 11, 12, 13, 14}. The number of fragments and the maximum number of atoms per fragment, 3 and 24, respectively, were chosen so as to include interactions between {H15, H16, H17} and {H42, H43, H44}. Without including these interactions, there would be no repulsion between these groups of atoms, which could result in spatial overlap between the two ends of the molecule. The three fragments are shown in the red boxes of Figure 4.

The final PIPs basis was obtained by running the MSA software^4,44^ for each fragment, with a maximum polynomial order of 2. Then the Mathematica-based software^64^ combines the three bases, deletes duplicate polynomials, and writes a Fortran file for use in fitting. The final basis has a modest size, i.e., 14,739 PIPs and linear coefficients.

### Many-Body Basis and PES

This basis is the union of 2-b, 3-b and 4-b bases, each with different Morse range parameters and distance cutoffs. Rather than optimizing these hyperparameters, they were set according to the general expectation that the *n*-body interactions fall off as *n* increases. For the 2-body, CH, HH, and CC Morse variables, a single range parameter of 2.5 bohr was used. Also a maximum power of 10 is used, so the total number of 2-body coefficients is 30. The cutoff distance for 2-body is 10 Å, that is, if the distance between two atoms is larger than 10 Å, that pair does not contribute to the total energy. Between 9 and 10 Å, a polynomial switching function^65^ is applied that damps that interaction to zero. For the 3-body, the Morse range parameter is 1.8 bohr, and the maximum polynomial order of the PIPs is 8. The switching function is applied when the maximum distance in a trimer is between 7 and 8 Å, and the energy contribution is 0 when the maximum internuclear distance in the trimer is beyond 8 Å. For the 4-body bases, the Morse range parameter is 1.2 bohr, and the maximum polynomial order is 7. The switching range for 4-body is between 5 and 6 Å. All the bases are purified,^66,67^ that is, any polynomial that does not go to zero when an atom is infinitely far away from the remaining atoms is removed from the bases. There are 32, 78, 78, 32, 77, 234, 354, 234, and 77 PIPs for CCC, CCH, CHH, HHH, CCCC, CCCH, CCHH, CHHH, HHHH, respectively. The final basis has 1227 terms, i.e., 30 (2-b) + 220 (3-b) + 976 (4-b) terms plus a constant, representing the total 1-body energy of 14 carbons and 30 hydrogens. This number of terms is an order of magnitude smaller than the number of terms in the fragmented approach. However, there are many terms to evaluate. Specifically, with the above choice of hyperparameters there are about 10^4^ 4-b interactions (the exact number depends on the geometry, and is typically between 10,000 and 25,000). Without a cutoff there would be 135,751 4-b interactions.

Finally, we note that this basis is manifestly fully permutationally invariant with respect to all permutations of like atoms.

## Results

We consider a number of results here, starting with the standard precision metrics for training and testing. Then we move to an examination of potential cuts, which include out-of-sample DFT data, and finally to extensive molecular dynamics calculations.

### Training and Testing Metrics

Results for precision of fits and selected direct comparisons with the training data set are given for the fragmented and many-body approaches. For both approaches computations were performed on a single node of a Linux cluster using a Xeon Gold 6250 processor with 128 GB of memory. The dgelss subroutine for linear regression in the Intel MKL library was used to obtain the expansion coefficients for the both the many-body PES (MB-PES) and Fragmentation PES (F-PES). This routine uses singular value decomposition to solve the well-known equations in overdetermined linear least-squares regression.^68^

For both the F-PES and MB-PES, fits were done using the data set shown in Figure 3. Inverse energy weighting was used in the F-PES, where the weights are given by 0.02/(0.02 + Δ*E*), where Δ*E* is the energy above that of the global minimum in hartrees.

Correlation plots for the F-PES vs B3LYP and MB-PES vs B3LYP training energies are given in Figure SI-3 over the range 0 to 80,000 cm^–1^. As usual, the precision deteriorates for energies at the high end of this range.

Precision metrics for the F-PES and MB-PES are given Table 1 along with a summary of the hyperparameters of the fits and the datasize. There are a two sets of metrics; one set is for the standard train/test split, with a 80:20 split selected. These are labeled by “split”. As seen, these eRMSEs are about the same for F-PES and MB-PES. This indicates that the PESs are not overfit. Precision metrics are given for fitting the full data set, labeled “no split”. These are close to the eRMSEs for the split data sets. The RMSE and MAE for the gradients for the fits to the full set of energies are given for a random set of 2000 configurations in the B3LYP data set. Finally the MAEs of harmonic frequencies of the 126 normal modes at the global minimum are also given.

**Table 1:** Precision Metrics for Fragmented-Basis and Many-Body PIPs Potentials

PES:	fragmented	many-body
fragmentation (atoms)	24,23,23	-
number of energies	253,646	253,646
number of coefficients	14,739	1227
eRMSE^a^ (cm^–1^) train split	195	91
eRMSE (cm^–1^) test split	262	103
eRMSE (cm^–1^) train no split	206	92
eWRMSE (cm^–1^) train no split	108	92
eMAE (cm^–1^) train no split	86	43
*R*^2^(*E*) train no split	0.999557	0.999911
gRMSE (cm^–1^/bohr) train no split^c^	422	216
gMAE (cm^–1^/bohr) train no split^c^	206	78
MAE frequencies^b^ train no split (cm^–1^)	25	7.6

a eRMSE: root-mean squared error; eWRMSE: weighted RMSE of energies; MAE: mean absolute error; e: energy; g: gradient.

b From normal-mode analysis at the global minimum.

c Gradients were not used in fitting the PES.

From these metrics it is clear that the MB-PES is more precise than the F-PES. However, as shown below, the F-PES runs much faster than the MB-PES, so that is a major asset of the F-PES, and we exploit that in extensive molecular dynamics calculations. Additional comments about these two fitting methods are given in the Discussion section.

Next, we examine the permutational symmetry of the F-PES. As noted above, the fragmented basis does not manifestly describe the permutational symmetry with respect to the interchange of all C and H atoms. Specifically, this symmetry exchanges the coordinates between C1 and C14, C2 and C13, ···, C7 and C8, as well as between H15 and H44, H16 and H43, ···, H29 and H30. However, the F-PES can “learn” this symmetry from the data set. To examine this, we took 2,000 geometries at random from the data set, changed the geometries by replacing the order in the *xyz* data with the interchanged order. We then calculated the energies for both the original and interchanged structures and compared them, as shown as a correlation plot in Figure SI-4. There is an excellent correlation despite the fact that the interchanged structures were not included in the original data set. The eRMSE is 282 cm^–1^, the MAE is 129 cm^–1^/bohr, and the *R*^2^ coefficient is 0.999112. The reason that the correlation is so good appears to be due to the fact that the data set is so large. While exact interchange symmetry is not a feature of the structures in the data set, enough similar structures are included so that the fit approximately maintains this symmetry.

**Figure 5:**
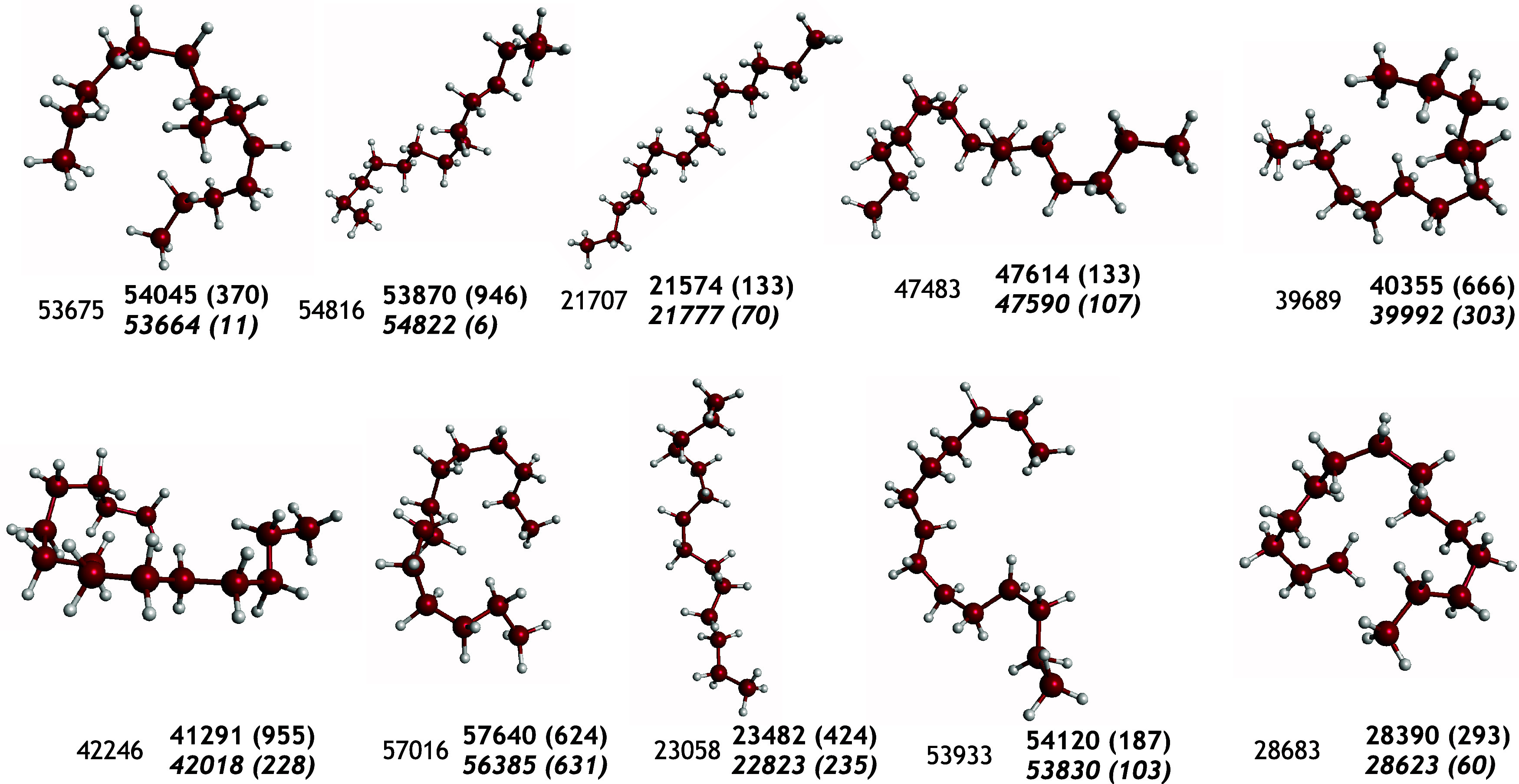
Ten selected structures from the data set and energies in cm^–1^ relative to the global minimum energy. The numbers in boldface are from F-PES, italic-boldface MB-PES and in regular font B3LYP values at the indicated configuration. Absolute differences in energies are indicated in paretheses.

Next we show comparisons of the PESs with ten samples from the training data set that illustrate the large span of structures and energies of C_14_H_30_ in Figure 5. As seen there, the energies from the fits for these samples are within 2% or less of the training energies for the F-PES and within 1% or less for the MB-PES. Clearly the fits are providing faithful representation of this high diverse and high energy data set.

## Beyond Precision

Once a satisfactory PES has been obtained, based on the usual precision metrics, it important to go beyond these metrics. We do that next.

### Potential Energy Cuts for Rotations Around C–C Axes

First, we examine cuts of the PES, both for testing the PES and for learning what controls the C_14_H_30_ configurations. Starting with the molecule in the GM configuration, we determined geometries for torsional rotation cuts around several C–C bonds by (a) shifting the center of rotation to one of the selected carbons, (b) rotating the C_14_H_30_ so that the selected C–C bond is in the *z* direction and so that another selected atom is in the *z*–*y* plane (this rotation is done by solving for the Euler angles and then using the Euler matrix to rotate the entire molecule), and then (c) rotating a piece of the molecule around the *z* axis by calculating for a series of torsional angles the *x* and *y* coordinates of each atom to be rotated.

**Figure 6:**
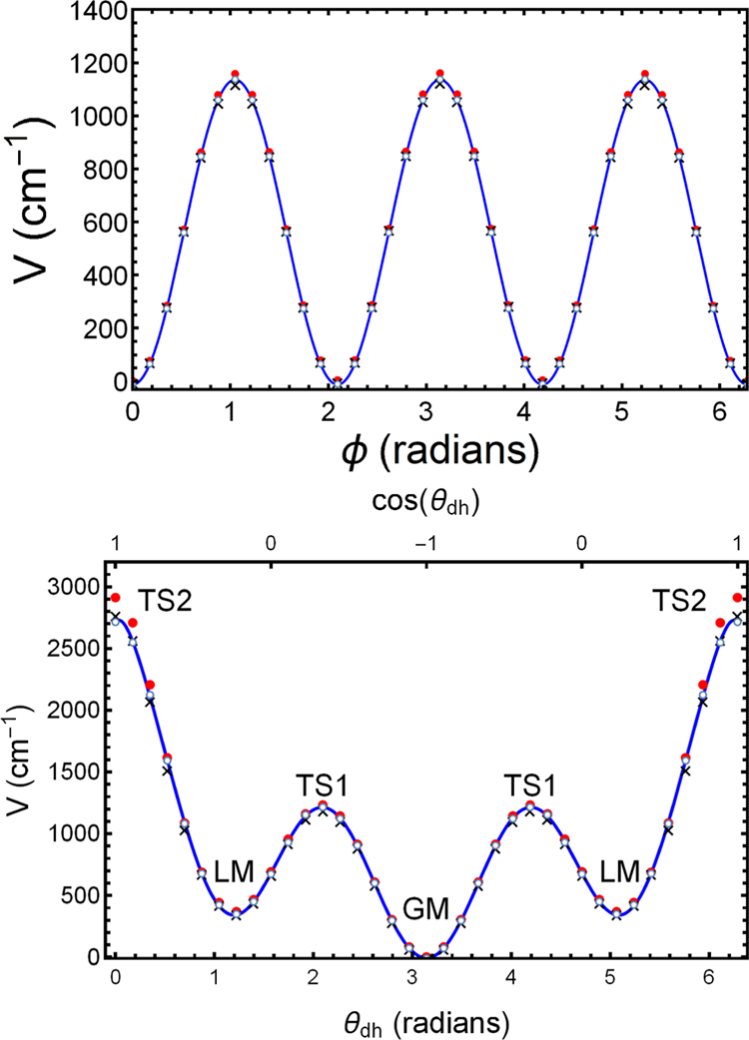
(Upper panel) Torsional potential (blue solid line fit to open-circle DFT data, unrelaxed) for methyl rotation around the C13–C14 axis. The red points are the predictions of F-PES, whereas the black × markers are the predictions of MB-PES. (Lower panel) Torsional potential (blue solid line fit to open-circle DFT data, unrelaxed) for rotation around the C12–C13 axis, showing the positions of the global minimum, the local minima, and the transition states. The red points are the predictions of F-PES, whereas the black × markers are the predictions of MB-PES. TS2 is the barrier to free rotation in which the C11–C12–C13–C14 are all in the same plane in a “u”-shape, whereas TS1 is the barrier between minima in the zigzag form (GM) and a partially rotated form (LM). The DFT energies are GM = 0 cm^–1^, TS1 = 1226 cm^–1^, LM = 359 cm^–1^, TS2 = 2725 cm^–1^.

The first example is for rotation of the C14-based methyl group around the C13–C14 axis. Once the geometries are determined by the above method, the PES is used to calculate the potential energy as a function of angle, as shown by the red dots in the upper panel of Figure 6. Shown also as open circles are the DFT energies. A fit to these DFT energies of the form sin(3ϕ – ϕ_0_) + *a* was then determined using the FindFit function in Mathematica. The best fit is shown by the blue line of the figure. It should be emphasized that this is an “unrelaxed” potential in the sense that the molecular configuration is fixed at the GM configuration except for the angle around the C13–C14 axis. It is to be expected that the potential energies will be somewhat lower if the molecule is allowed to change (relax) its geometry. (One dimensional eigenvalues, obtained using the Colbert-Miller Discrete Variable Representation (DVR),^69^ were obtained and the results are shown in Figure SI-5.)

The next example is a PES cut for rotation around the C12–C13 axis shown in the lower panel of Figure 6. Again, the red dots represent the energies obtained from the PES for the unrelaxed torsional geometries, while the blue line is a fit to the DFT-calculated energies using a function of the form *a*_0_ + *a*_2_(θ_dh_ – π)^2^ + *a*_4_(θ_dh_ – π)^4^ + *a*_6_(θ_dh_ – π)^6^ + *a*_8_(θ_dh_ – π)^8^ + *a*_10_(θ_dh_ – π)^10^ + *a*_12_(θ_dh_ – π)^12^. The angle of rotation is the dihedral angle between the plane containing C11, C12, and C13 and the plane containing C12, C13, and C14. Labels identify the GM, a local minimum (LM), a transition state (TS1) between the GM and LM and a higher transition state (TS2), above which there is free rotation.

We comment briefly on the torsional potential around the C12–C13 bond shown in the lower panel of Figure 6. There are two unusual features. The GM has a zigzag structure; the dihedral angle for such a structure is π. One might have thought that the “u”- or boat-shaped structure would be another minimum, but we find that it is actually a transition state (TS2) with a fairly high barrier, 2931 cm^–1^, for the unrelaxed structure. The reason is that the hydrogen atoms interact with one another in an unfavorable way, so that the LM structures are more stable where the dihedral angles are not 0 and 2π but rather about 1.1 and 5.05 radians. The second feature of the potential is that TS1 is not, as might have been expected, at a θ_dh_ of π/2 and 3π/2 but rather at about 2.05 and 4.1 radians.

We also examined rotation around the C7–C8 axis and found a potential that was, to within a few cm^–1^, identical to that shown for rotation around the C12–C13 axis.

We further examined unrelaxed rotation around C12–C13 and around C9–C10 starting from the LM for rotation around C7–C8. In this case, the energy profile looked much like that in the lower panel of Figure 6 except that the origin was shifted upward by about 359 cm^–1^. It appears that the energies for the C12–C13 rotation are just added to those for the C7–C8 LM.

A summary of the energies of stationary points for the methyl torsion and C12–C13 torsion is provided in Table 2. Briefly, the unrelaxed F-PES and MB-PES do well compared to the unrelaxed DFT benchmark. The unrelaxed MM3 calculated values are shifted substantially (ca. 430 cm^–1^) in energy, based on the DFT GM, but after introducing this shift the MM3 table results predict TS1 in good agreement with the DFT benchmark. However, the predictions for LM, TS2, and the methyl torsion TS are all considerably low. The optimized F-PES and MB-PES are in good agreement with the relaxed DFT benchmark.

**Table 2:** Energies of Stationary Points on F-PES, MB-PES, MM3, and Direct DFT (in cm^–1^)

stationary point	LM	TS1	TS2	methyl torsion TS
F-PES (unrelaxed)	368	1232	2912	1158
MB-PES (unrelaxed)	359	1201	2780	1130
fit to F-PES (unrelaxed)	361	1238	2931	1155
DFT Benchmark (unrelaxed)	359	1226	2725	1148
MM3^a^ (unrelaxed)	307	1224	2288	997
optimization on F-PES	309	1140	2013	1080
optimization on MB-PES	301	1135	1963	1071
DFT benchmark (relaxed)	304	1165	2004	1091

a After shifts in phase and GM energy.

### Molecular Dynamics Calculations

Another way to examine the F-PES and MB-PES is via molecular dynamics (MD) calculations, which we did using our own software.^70,71^ Owing to the difference in speed of evaluation, most calculations were done with the F-PES.

Specifically, microcanonical (NVE) trajectories were run using F-PES and employing a time step of 5 au, or about 0.121 fs for 10,000 steps. In all applications trajectories were initiated at the global minimum of the potential and the total energy was specified and distributed uniformly among the kinetic energies of all atoms. The total angular momentum was adjusted to be zero for each trajectory. Properties were obtained at total energies of 5000, 15,000, 25,000, 35,000, 50,000 and 80,000 cm^–1^. At each energy batches of trajectories were propagated with a recording of the energy, geometry, velocities, and gradients at every 10 steps.

Figure SI-6 shows the F-PES potential energy distributions for the points in the trajectories where recordings were made. As expected from equipartition, the potential energy distribution peaked at one-half the total energy. Also shown in the figure are the distributions for the MM3 trajectories at 500 and 1000 K that formed part of the data set. The correspondence between temperature and total microcanonical energy is approximately 50,000 cm^–1^ ≈ 570 K and 80,000 cm^–1^ ≈ 900 K.

A property of interest for this flexible hydrocarbon is the end-to-end distance, i.e., the C1–C14 distance, as a function of the total internal energy. Before we show those, we discuss some important details of these calculations. First, we needed to determine if 10,000 steps are sufficient to obtain stationary results for this property, especially given the large range in total energy. This was done by recording the distribution vs the time of the trajectory at every 10th time step and examining the distribution vs the time. An example of this is shown in Figure SI-7 for 50,000 cm^–1^, and the distribution does appear converged at 9000 and 10,000 steps. Second, we kept track of failed trajectories, which are manifested by an “exploding” molecule. These occur in regions of configuration space not within the training data set; these are typically in high-energy regions, where the machine-learned potential has “holes”, i.e., large negative values. This is a ubiquitous aspect of machine-learned potentials as reviewed in some recent papers.^72,73^ Of the 100 trajectories run at 5,000 and 15,000 cm^–1^, there were no exploding trajectories, for 25,000 cm^–1^ there were 2 and at 35,000 cm^–1^ there were 10. These are small or zero numbers of failed trajectories and so there are a sufficient number of completed trajectories to obtain the C1–C14 distance distribution. However, at 50,000 and 80,000 cm^–1^ more exploding trajectories were found, as expected. As a result, we ran more trajectories. For 50,000 cm^–1^, we ran 400 trajectories, of which 240 completed. For 80,000 cm^–1^, we ran 700 trajectories, of which 154 completed. These were used to obtain the distribution of the C1–C14 distances.

**Figure 7:**
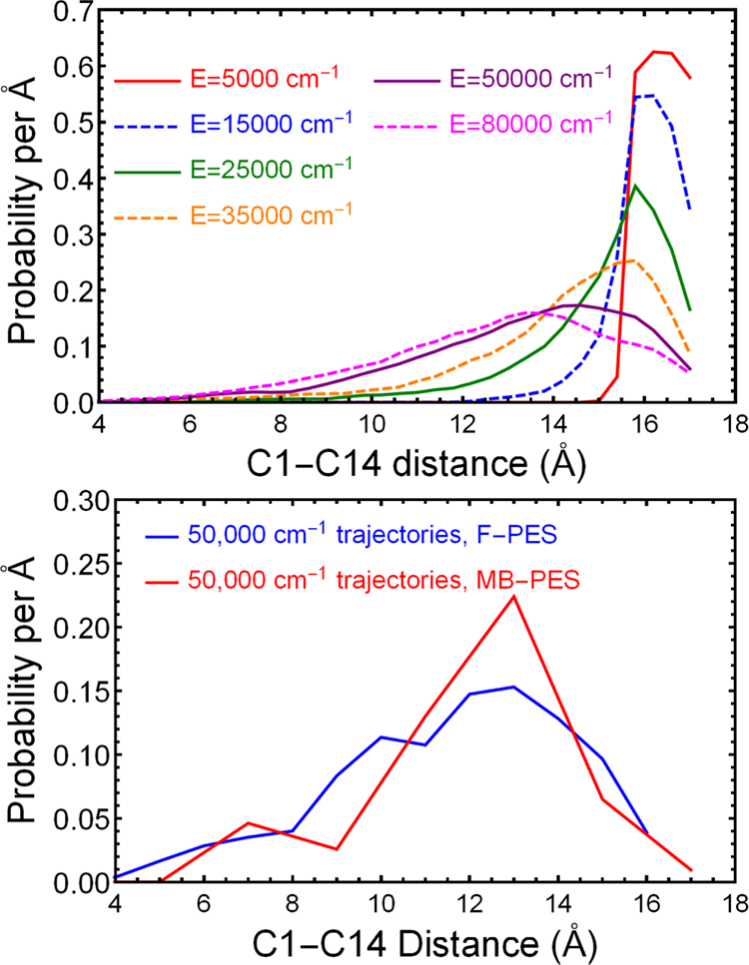
(Upper panel) Distribution of the C1–C14 internuclear distances for entire F-PES MD trajectories run at the indicated total energies starting from the GM and propagated for 10,000 steps (1.21 ps). (Lower panel) Comparison of a C1–C14 distance probability distributions evaluated from the final 1000 trajectory steps of the fragmented (blue) and many-body (red) trajectories at *E* = 50,000 cm^–1^. Probabilities per Å are the probabilities per bin divided by the bin width in Å.

Results for the C1–C14 distribution using data from entire trajectories are shown in upper panel of Figure 7. The distribution broadens with energy, and the higher energy trajectories have distributions shifted to lower C1–C14 distances. Qualitatively, this means that the C_14_H_30_ bends back on itself more easily at high energies, whereas at low energies it is more restricted to the linear zigzag shape, as expected. (We show selected snapshots below from an 80,000 cm^–1^ trajectory; this illustrates this bending.) The approach of discarding failed trajectories does produce very reasonable and expected results.

To further examine the robustness of the above results, we performed a similar analysis using the MB-PES at 50,000 cm^–1^. This was done for only five trajectories, owing to the much larger computational effort in MD calculations with MB-PES. In this case, for both the F-PES and MB-PES trajectories, the distance was recorded at every 10th step for the last 1000 steps of the trajectories. The results, shown in the lower panel of Figure 7, are in good agreement with each other. Furthermore, the distributions are similar to the one for 50,000 cm^–1^ shown in the upper panel. We conclude that both PESs provide reasonable results.

**Figure 8:**
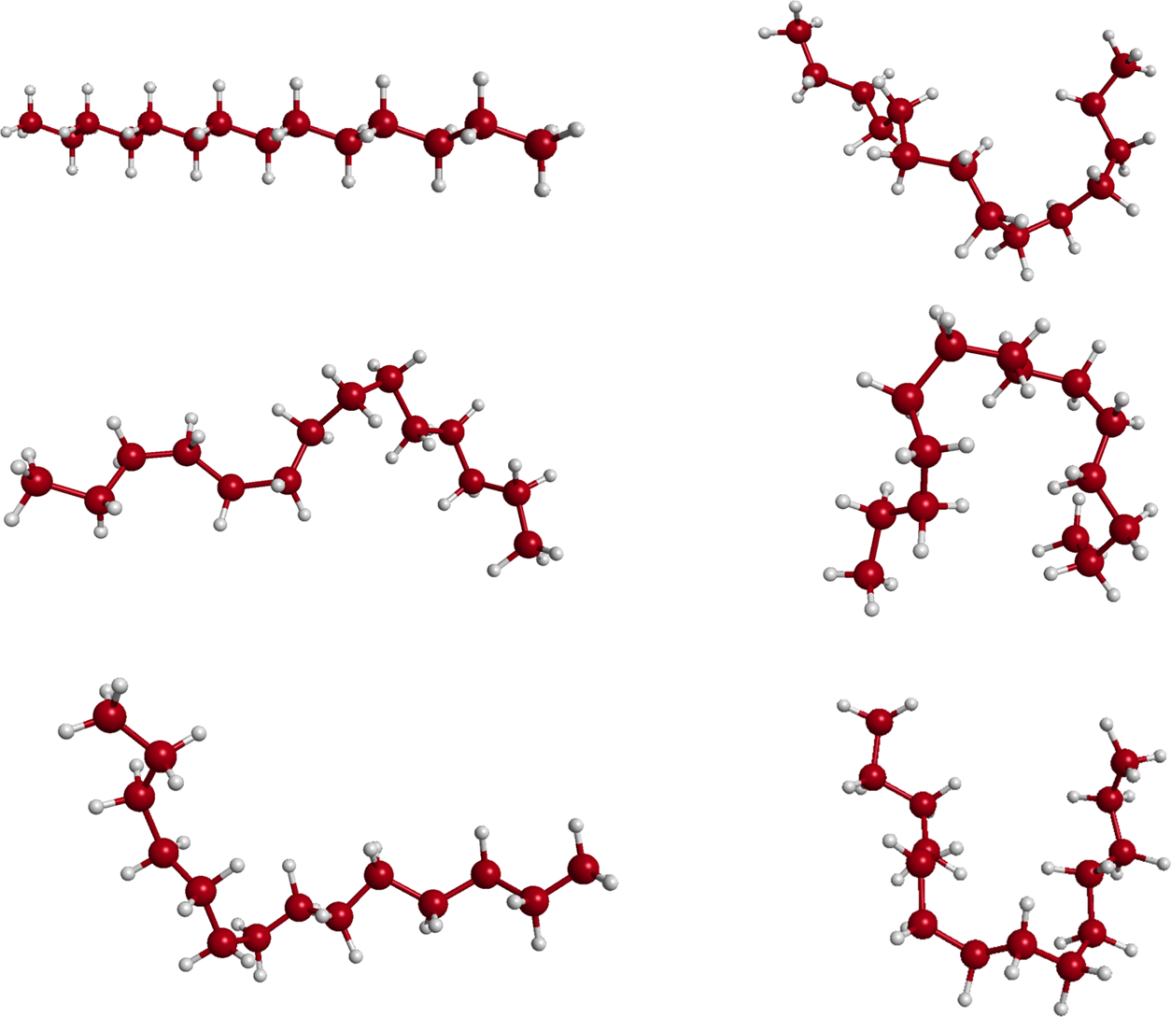
Snapshots of an MD trajectory using fragmented PIP basis PES at 80,000 cm^–1^.

To indicate the variety of configurations described by the PES, we show in Figure 8 snapshots of molecular shapes from one trajectory of total energy 80,000 cm^–1^. The linear configuration corresponds to the initial condition at the global minimum. Clearly, the shape changes dramatically along this trajectory and this illustrates the ability of the PESs to describe this flexibilty.

**Figure 9:**
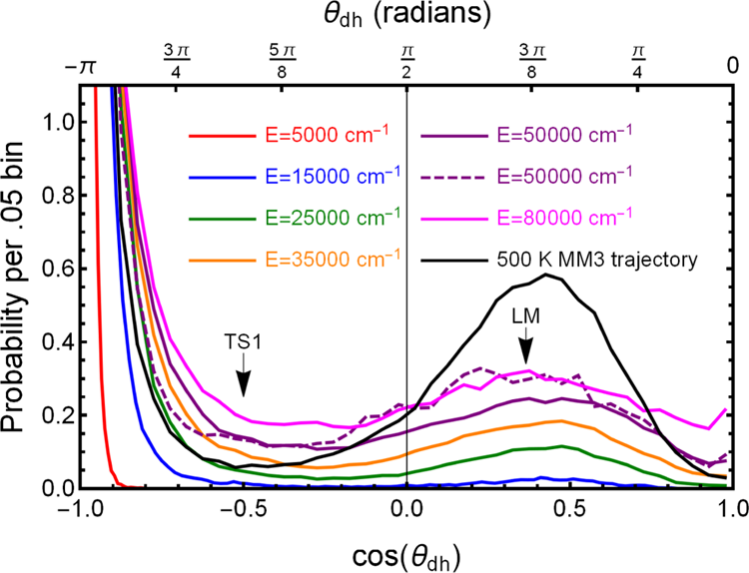
Solid colored lines show histograms of the dihedral angle distribution around all C–C bonds at all geometries for F-PES MD trajectories run at various total energies starting from the GM. Higher energy trajectories reach more positive values of cos(θ_dh_), meaning that there are fewer zigzag geometries. The result for the MB-PES trajectory at 50,000 cm^–1^ is given by the dashed purple line, while the result for the MM trajectory at 500 K is given in the solid black line. The cos(θ_dh_) values for the GM and TS2 are −1 and 1, respectively; the values for TS1 and LM are marked.

We next examine the flexibilty of rotation around the C–C bonds of C_14_H_30_. Figure 9 shows the F-PES distributions of all dihedral angles and all trajectory recorded geometries for each of the six energies. The distribution has two peaks, a strong one at cos(θ_dh_) = −1, the value corresponding to the GM, and a subsidiary peak at the value corresponding to the LM. We note that the higher the energy, the more population there is in the LM. The 50,000 cm^–1^ MB-PES trajectory result was similar to that of the F-PES result except that there was slightly more population at positive cos θ_dh_, as shown in the dashed purple line. The figure also shows (in the black line) the distribution for the 500 K MM3 trajectory used in the data set. It is quite different from the distributions for the PES trajectories, despite the fact that the energy distributions for the 500 K MM3 and for the 50,000 cm^–1^ trajectory are quite similar. Because the MM3 and DFT energies do not agree, this is not too surprising. Finally, it is interesting to note that evaluation of energies by MM3 using Tinker is no faster than the much more accurate evaluation using the fragmented basis set. In addition, F-PES provides relatively fast gradients, if desired.

### Timing

Timing evaluations for 100,000 energies and gradient sets were performed on a 2.3 GHz Intel Xeon Gold 6250 core. For F-PES, the results are 5.62 and 21.01 s, respectively, using reverse differentiation for the gradient sets. Note that the ratio of gradient to energy time is about 3.75, consistent with Figure 6 of ref (19) which indicated that for reverse differentiation this time ratio is independent of the number of atoms in the molecule. For the MB-PES, the times are approximately 100 times higher. This is mainly due to the large number of 4-b interactions. It is noteworthy and not surprising that the F-PES and MB-PES are roughly 6 and 4 orders of magnitude faster, respectively, than the B3LYP calculation of the electronic energy and forces, which costs about 320 s per geometry, on a single Intel Xeon Gold 6250 core. It is, however, perhaps surprising that F-PES runs only 4 times slower than the MM3 force field (Table 3).

**Table 3:** Time for Calculations of Energies and Gradients for 100,000 Geometries for C_14_H_30_

PES/measurement	energies	gradients	energies and gradients^a^
F-PES^b^	5.62 s	21.01 s	21.01 s
MB-PES^b^	581 s		2153 s
MM3^c^			5.07 s
MM3^d^			8.54 s

a Evaluated simultaneously.

b Intel Xeon Gold 6250, 2.3 GHz.

c Apple M1 Pro, 3.2 GHz.

d AMD 3900X, 3.8 GHz.

## Discussion

To begin this section, consider the data set presented here. In the context of popular data sets for MLPs, e.g., rMD17^29^ the DFT data set presented here for C_14_H_30_ is probably the most extensive one reported. The energy range used for the fits extends from near zero (the global minimum) to more than 80,000 cm^–1^ (229 kcal/mol). In addition, the data set spans a very large range of configurations of this alkane. This is of course a consequence of the well-known flexibility of alkanes. The extensive range of energies and configurations, as well as the large number of atoms, present an opportunity to test various machine-learning methods mentioned in the Introduction and the plethora of new ones. We have previously critiqued the rMD17 data sets as basically being limited to the lowest energy minimum of a potential.^59^ This is due, at least in part, to the sampling in that data set from MD simulations at 500 K. For 21-atom aspirin this limits the maximum potential to roughly 14,000 cm^–1^.^28^ By contrast, the data set used here comes from MD simulations at 500, 1000, and 2500 K, leading to a maximum potential value of 80,000 cm^–1^ for the training data set. (On a per atom basis this translates to 667 cm^–1^ for aspirin and 1818 cm^–1^ for C_14_H_30_.) Clearly the interesting science related to large molecules is the complexity of the energy landscape which exhibits numerous low-lying minima (and of course barriers separating them) and future studies of these, using the current PESs will be done.

As an aside, we note that the B3LYP energies (and gradients) in the data set do not account for long-range noncovalent interactions. Given the size of this molecule these could be significant, as stressed recently for model “toy” carbon chains,^74^ and, as noted above, by adding a classical many-body (for 5 and higher-bodies) polarization term for *ab initio* energies, by Paesani and co-workers for small hydrocarbons.^9^ Future work could include augmenting the present energies by long-range interactions.

Two different ML methods, both based on PIPs, were successful in fitting the data set of energies. The fragmented-basis approach uses a PIPs basis that is the union of three bases for fragments of 24, 23, and 23 atoms. This PIPs basis has 661 Morse variables compared to 946 variables (*N*(*N* – 1) /2), for this 44-atom molecule. Perhaps a single PIPs basis would be feasible for C_14_H_30_; but we did not pursue that. The main reason for that is it is clear that eventually a single PIP basis would not be practical for large molecules. We note that a single full basis was recently applied to 21-atom aspirin and shown to be very precise and much faster than many other ML methods.^28^ Instead, we applied the fragmented-basis approach, which was reviewed above already. As noted, there are many possible ways to fragment the basis, but here we reported results using a 3-fragment basis of size 24, 23, and 23 atoms. These are about the number of atoms in aspirin, where a single PIPs was used. Clearly the sum of the number of atoms is almost twice the number of atoms in the molecule. This is a consequence of the overlapping basis, i.e., a number of Morse variables are in common in the three PIPs bases. This can lead to duplication of PIPs in the final basis; however, these can be deleted using the software PESPIP.^5^

Details of the software used for the fragmented PESs are given, including a flowchart describing the freely available PESPIP software.^5^ So, we only briefly review the highlights here. MSA software^44,45^ is run first to generate the PIPs for any molecule with a user-specified permutational symmetry and also total polynomial order. The number of PIPs on output depends in a complex way on the permutational symmetry (the higher the former the lower the number of PIPs), the number of atoms and the total polynomial order.^3^ This basis of PIPs can be reduced with a Mathematica notebook, based on a user-defined size, and a sort method that orders the magnitude of each PIP over the configuration space of the data set. The fragmented basis is also generated using PESPIP, and this does require a user-defined process described here.

The software used for the MB-PES is still under development, and so is not yet freely available. However, the components are. Namely, the PIPs for each *n*-body term can be and were obtained using MSA. The least-squares fitting software, dgelss, is also used in the MSA software. However, the user would have to modify the call to that software, after assembling all the *n*-body terms. We intend to report more details about the software in the future.

Some comments about the permutational symmetry of the F-PES and the MB-PES are worthwhile. The MB-PES is inherently fully permutationally symmetric. This should be clear by simply noting that the order of summation over the C and H atoms for each *n*-body term is arbitrary and so invariant with respect to permutation of indices. A reasonable question to ask is whether this complete permutational symmetry is needed, when in fact H and C atoms do not exchange, at least at the energies of our data set. The answer is not straightforward for this highly atom-symmetric molecule. To see this, imagine a change in energy due to a small change in the bond distance between C14 and H44. If we were to instead perform a similar geometry change to the bond distance between C1 and H15, we should get the same change in energy. The permutational symmetry of the MB-PES ensures that this will be the case. However, the permutational symmetry of the F-PES is more nuanced. We know that the symmetry of the three H atoms in the two methyl groups is an essential symmetry, independent of the molecular configuration, as is the permutational symmetry of the pair of hydrogens on each carbon atom. Both of these symmetries are described in the F-PES. What is not explicitly described in the F-PES is the interchange symmetries of carbons; e.g., C1 with C14, C2 with C13, etc. Nevertheless, from the results of calculations shown in Figure SI-4, we see that this symmetry is obeyed numerically. This is because the data set contains configurations that are close to the interchanged ones.

Next, we comment briefly on the connection of the present MB-PIPs approach to earlier work using MB-approaches for molecular potentials. The earliest work for atomic solids, notably Si, dates back to the mid 1980s.^48,49^ For chemical reactions a MB approach was applied for three and four-atom systems, by Murrell and co-workers.^56^ Our early work with PIPs used a 2-b and then full-body decomposition for H_5_O_2_^+^,^75^ and we also noted the formal decomposition of a full PIPs basis into 2-b, 3-b, etc. PIPs terms.^3^ More recent and relevant, many-body, atom-centered ML methods have appeared, mainly for materials applications.^17,18,76−78^ The atomic cluster expansion^76^ (ACE) uses an atom-centered expansion, where interactions are 2-b, 3-b, etc. The basis for these *n*-body terms are given in terms of internuclear distances and angles and overall are more complex than the PIPs bases we use here, which are just functions of the transformed internuclear distances (Morse variables). The permutational invariance is directly built-in to PIPs whereas the procedure to build this invariance into the ACE basis is more complex. A somewhat related approach uses a basis of B-splines in an atom-centered expansion up to 3-b terms and applied to tungsten.^77^ This basis is fast to evaluate; however, it is not manifestly permutationally invariant. The method that is perhaps closest to one developed here is a successor to our PIPs approach and is termed aPIPs.^17,18^ The method was successful in terms of precision and speed relative to other ML MB approaches. However, it was not pursued further by those authors. Finally, we note that while the aPIPs, ACE and present MB-PIPs methods are polynomial linear regression methods, the former two belong to the class of underdetermined least-squares, i.e., more linear coefficients than data, whereas MB-PIPs (and all of our PIPs fits) are overdetermined, i.e., fewer linear coefficients than data. Another important difference is a formal one, namely aPIPs and ACE represent the total energy as the sum of atomic energies instead of the MB expansion given above.

Next, consider the large difference in evaluation speed of energies and gradients between the MB and F-PESs. While the MB-PES has a smaller number of linear coefficients than the F-PES, there are many more terms to evaluate in the former. By contrast, the evaluation of the F-PES is “once and done”. All MB approaches are hampered by the number of terms to evaluate and, as noted, this number is given by as  which goes as *O*(*N*^*n*^/*n*!). However, by applying a physically reasonable cutoff for each *n*-body term the actual number is far less. The cutoff recognizes that at long-range the *n*-body terms can be understood from multipole expansions and, notably, from many-body polarization. In the present work we did not examine in detail the effect of reducing the cutoff for the 4-b term and thereby reducing the number of 4-b terms. This is certainly something we plan to examine in detail in follow-up work. In addition, it is clear that the MB-PES can exploit straightforward parallelization which can also increase the speed of evaluation. Both aspects have been examined for our MB q-AQUA water potential^15,52^ and the interested reader is referred to those references for details.

The current MB approach has one great advantage over the fragmented-basis one, already noted; namely obvious transferability to larger “linear” alkanes. The transferability of the F-PES is not obvious; however, it does appear that the fragmented basis can be built up for larger hydrocarbons. However, it is not clear that the optimized coefficients for C_14_H_30_ can be reused for larger hydrocarbons. We intend to investigate this important aspect of these PIPs approaches in the future

Finally, we note that the current study demonstrates some of the strengths and weaknesses of the widely known MM3 force field.^61^ There is a substantial discrepancy between the MM3 energies and the DFT/B3LYP energies, as shown in Figure 1. In addition, we see from Table 2 that the energies of the stationary points LM, TS1, TS2 and the Methyl torsion TS are considerably low compared to those on either F-PES or MB-PES and, more importantly, compared to the DFT benchmarks. Finally, Figure 7 shows that the results from MM3 are not in good agreement with calculations of the C1–C14 distance distributions and of the dihedral angle distributions using F-PES or MB-PES. Clearly, the present machine-learned potentials can be used to assess and perhaps improve this and more recent^79^ classical force-fields for hydrocarbons. This is a fruitful area for future research.

## Summary and Conclusions

A large data set of B3LYP energies and gradients that spans a large configurational space has been reported for C_14_H_30_. Two linear regression PIP fits to a data set of 253,646 energies were reported. One uses a three-fragment approach consisting of 24, 23, and 23 atoms with a total polynomial order of 2. After removing duplicates, there are 14,739 terms in the PIP basis. This results in a modest linear algebra problem to determine the corresponding number of coefficients. The second approach is a many-body one, up to 4-b terms. This approach leads to a much small linear algebra problem to determine only 1227 coefficients. Both PESs produce precise fits and were shown to be robust for describing the torsional motion of this molecule and also for molecular dynamics calculations.

Finally, we hope that the data set presented here will be used to test other ML methods for representing large molecule potentials.

## Data Availability

The PESs used in this study are available by contacting the authors. The data set of B3LYP energies and gradients are available at https://github.com/jmbowma/QM-22/

## Supporting Information Available

Relative B3LYP/cc-pVDZ and MM3 energies at *T* = 500, 1000, and 2500 K; scatter plot of B3LYP/cc-pVDZ and MM3 energies vs the C1-C14 distance at *T* = 500, 1000, and 2500 K; correlation plot showing energies and cumulative RMS error, calculated using F-PES as a function of ab initio (B3LYP) training energy; plot of the predicted energies for 2000 geometries chosen at random from the MD data set showing the correlation between the energies of the original geometries and those for the energies of the interchanged geometries (PDF)
