# Supplementary material for: DFT-Based Permutationally Invariant Polynomial Potentials Capture the Twists and Turns of C14H30
Source: J Chem Theory Comput. 2024 Oct 21;20(21):9339–53. doi: 10.1021/acs.jctc.4c00932 (PMC11562071; doi:10.1021/acs.jctc.4c00932)
Supplement: Supplementary file 1 — ct4c00932_si_001.pdf [file ct4c00932_si_001.pdf]

Supporting Information: DFT-Based Permutationally Invariant Polynomial Potentials Capture the Twists and Turns of $C_{14}H_{30}$

Chen Qu,[†] Paul L. Houston,[*,‡] Thomas Allison,[¶] Barry I. Schneider,[§] and Joel M. Bowman[*,‖]

†*Independent Researcher, Toronto, Ontario M9B0E3, Canada*

‡*Department of Chemistry and Chemical Biology, Cornell University, Ithaca, New York 14853, U.S.A. and Department of Chemistry and Biochemistry, Georgia Institute of Technology, Atlanta, Georgia 30332, U.S.A*

¶*NIST, Gaithersburg, MD 20899 USA*

§*NIST, Gaithersburg, MD*

‖*Department of Chemistry and Cherry L. Emerson Center for Scientific Computation, Emory University, Atlanta, Georgia 30322, U.S.A.*

E-mail: plh2@cornell.edu; jmbowma@emory.edu

Data Set Histograms

Histograms of energies are shown in Fig. ?? for 500 and 1000 K MD simulations. The lower panel shows these from the MM3 direct-dynamics calculations and the upper panel shows the corresponding DFT energies. As seen, they are in semi-quantitative agreement. However, only the DFT energies are used in the fits.

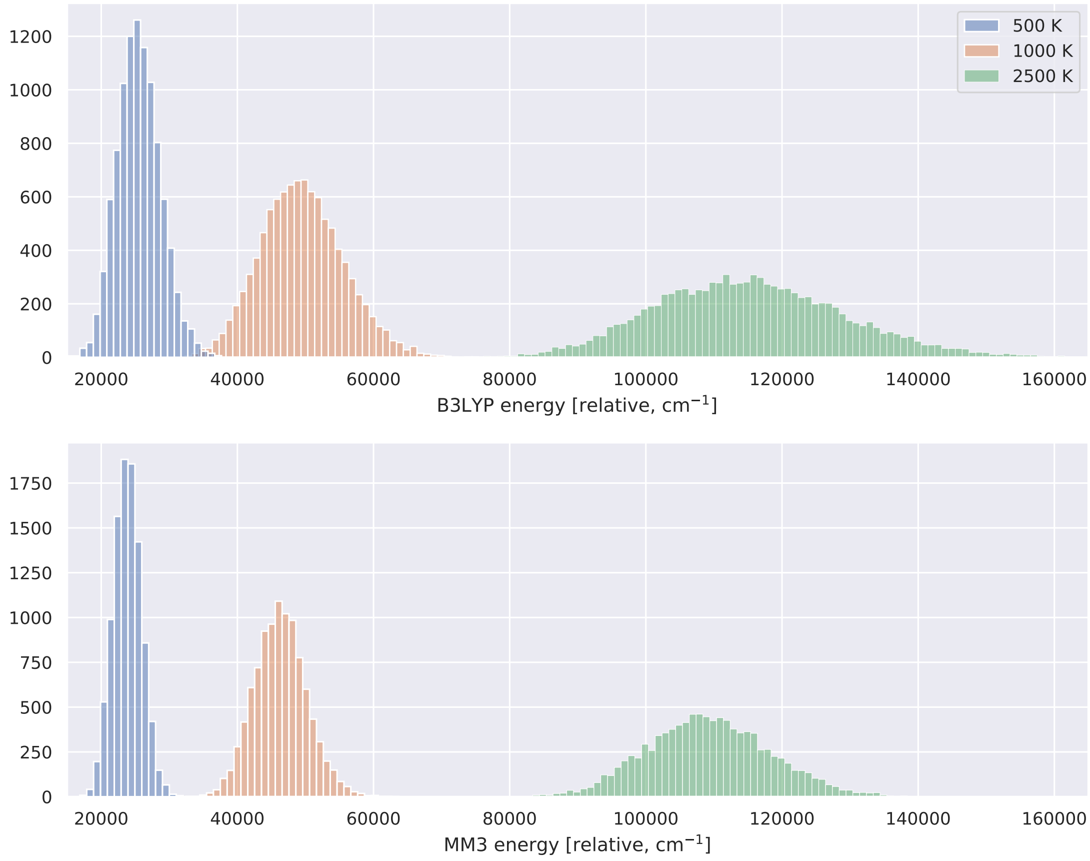

Figure SI-1: Relative B3LYP/cc-pVDZ and MM3 energies at T = 500 K, 1000 K and 2500 K.

Next we show relative B3LYP/cc-pVDZ and MM3 energies as a function of the C_1-C_{14} distance and distributions of both, at the temperatures indicated, in Fig. ??.

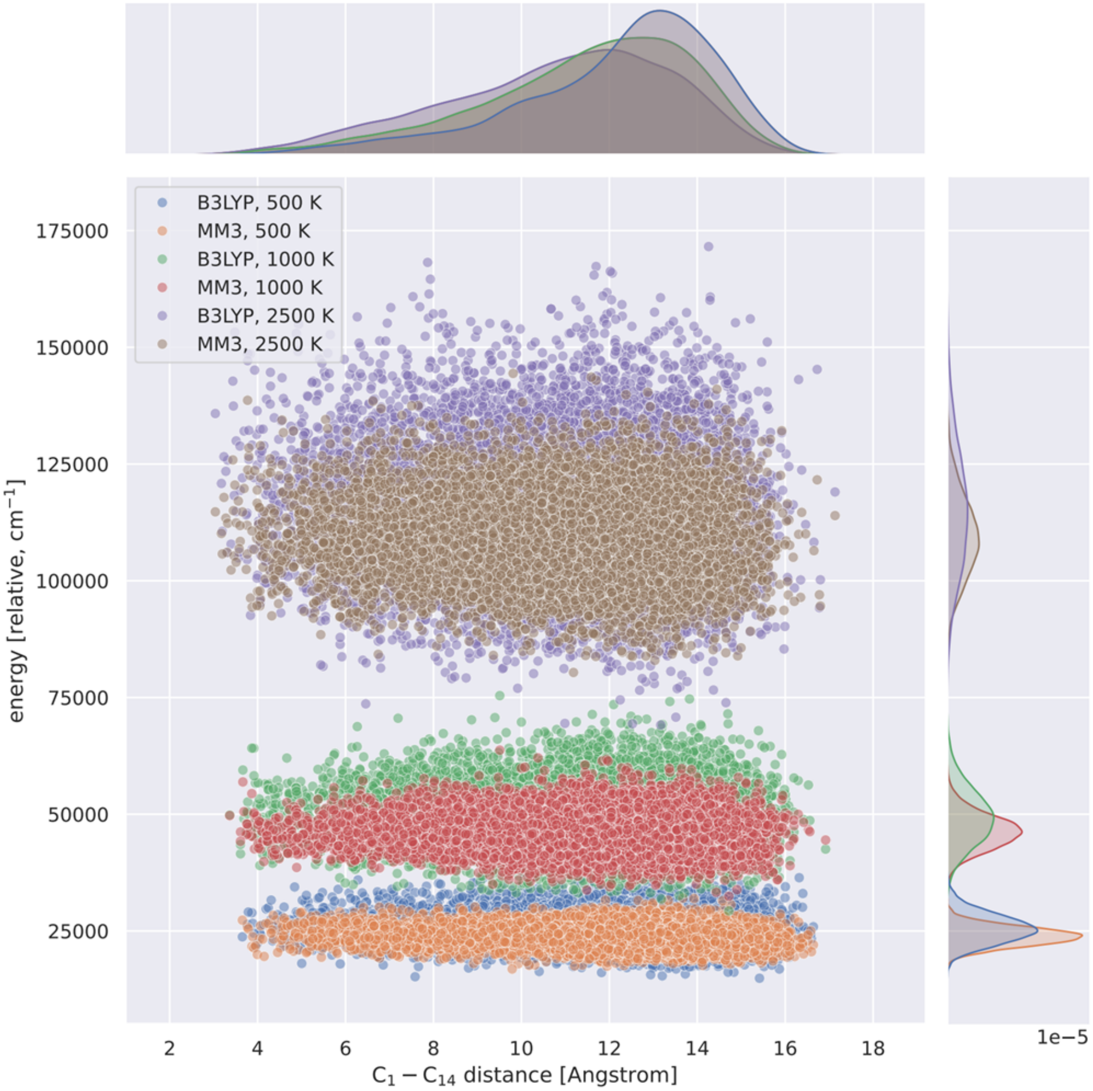

Figure SI-2: Scatter plot of B3LYP/cc-pVDZ and MM3 energies vs the C_1-C_{14} distance at T = 500 K, 1000 K, and 2500 K.

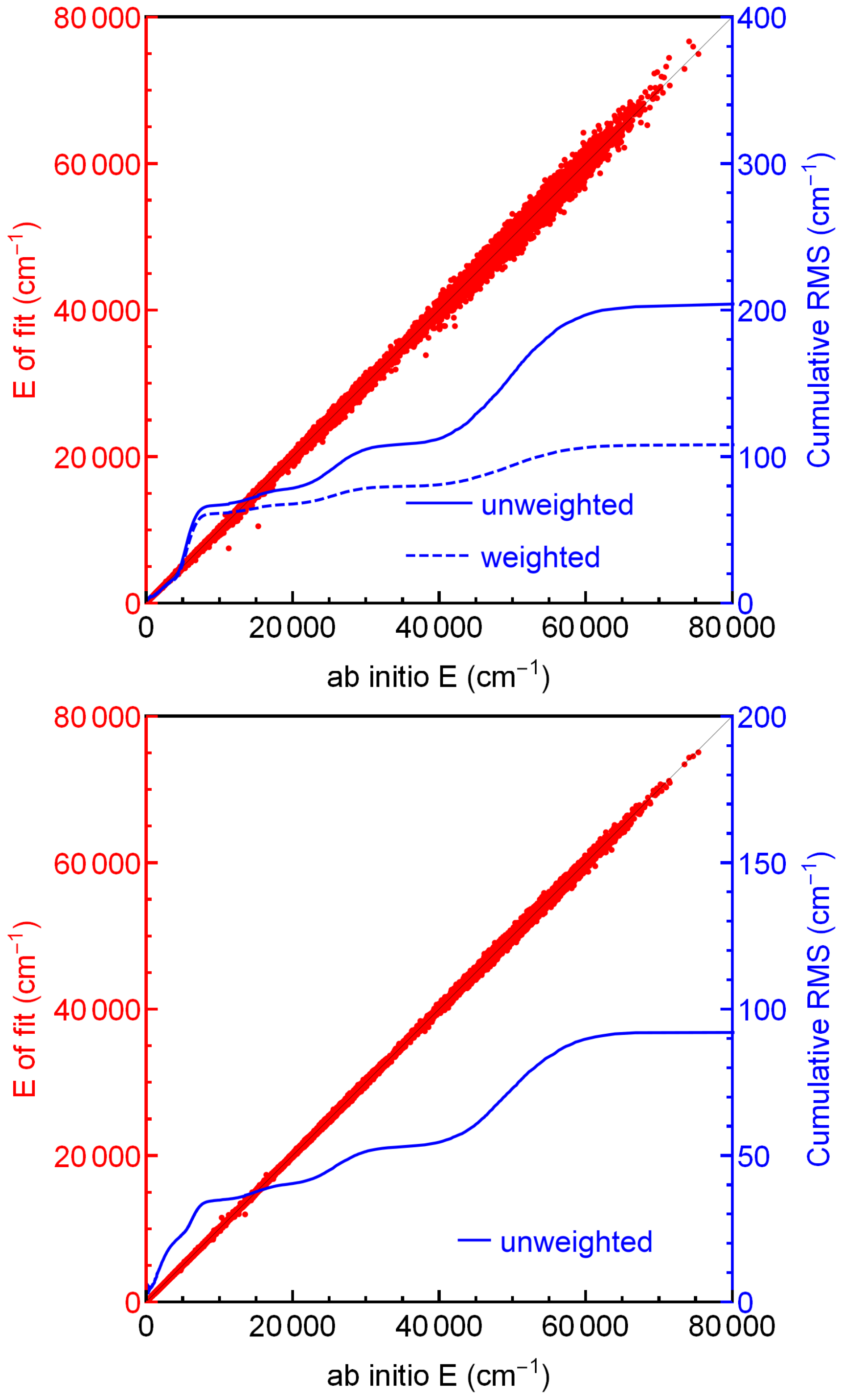

Figure SI-3: (Upper) correlation plot showing energies and cumulative RMS error, calculated using F-PES as a function of *ab initio* (B3LYP) training energy; (lower) correlation plot showing MB-PES energies vs. B3LYP training energies.

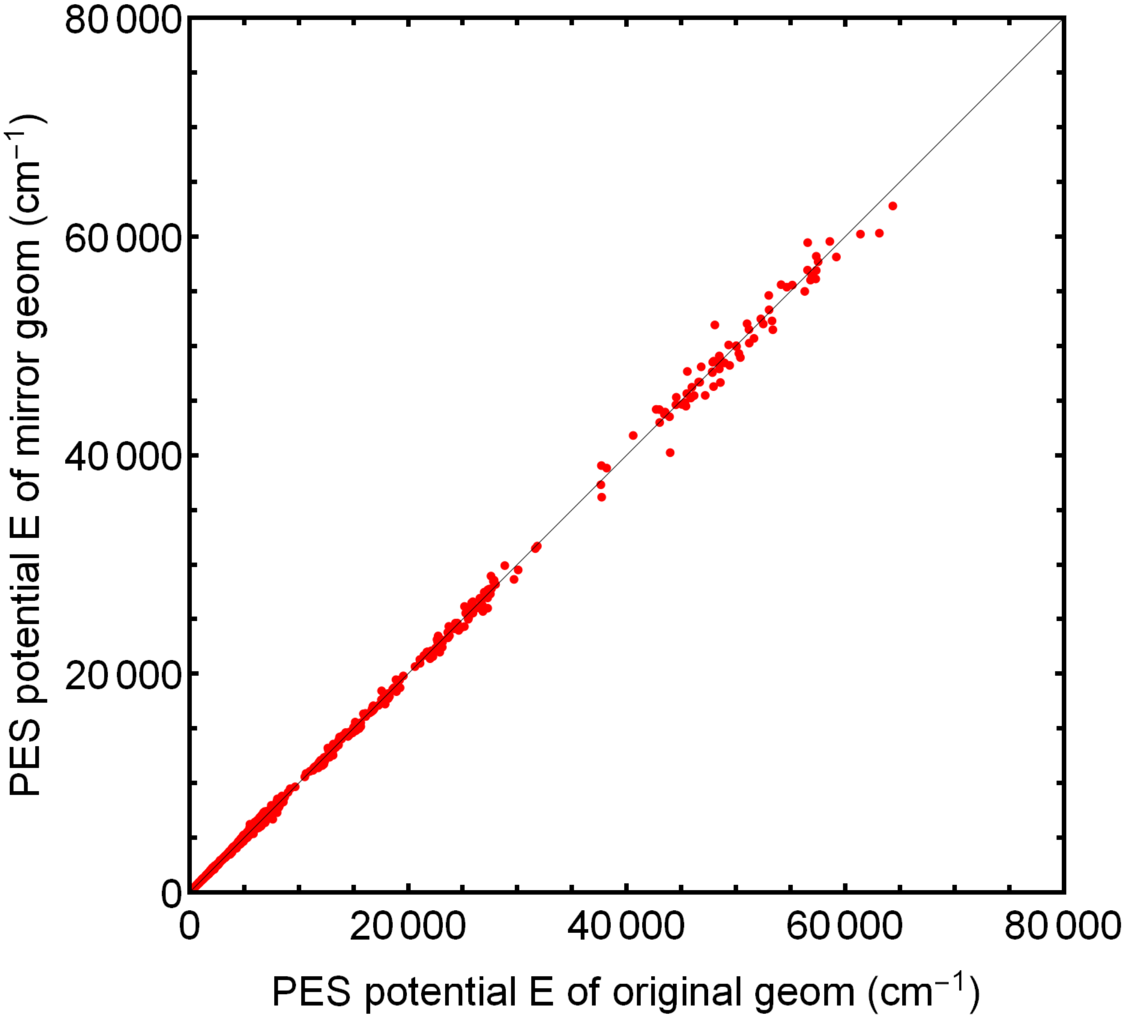

Figure SI-4: Plot of the predicted energies for 2000 geometries chosen at random from the MD data set showing the correlation between the energies of the original geometries and those for the energies of the interchanged geometries. The interchanged geometries were not used in the PES fit.

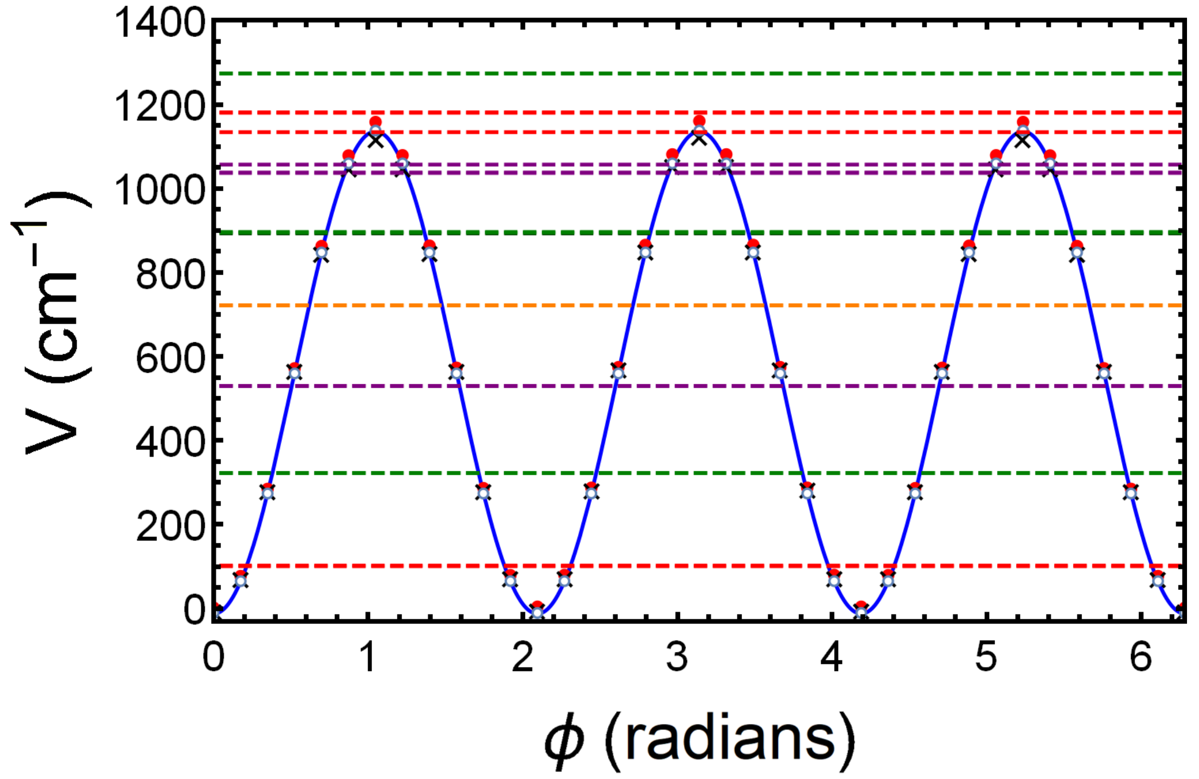

Figure SI-5: Torsional potential (blue solid line fit to open-circle DFT data, unrelaxed) for methyl rotation around the C13-C14 axis. The red points are the predictions of F-PES, whereas the black $\times$ markers are the predictions of MB-PES. The dashed lines give the energy levels calculated by DVR assuming that the moment of inertia is that of the methyl around the C12-C14 axis. The DFT torsional barrier is 1148 cm^{-1}.

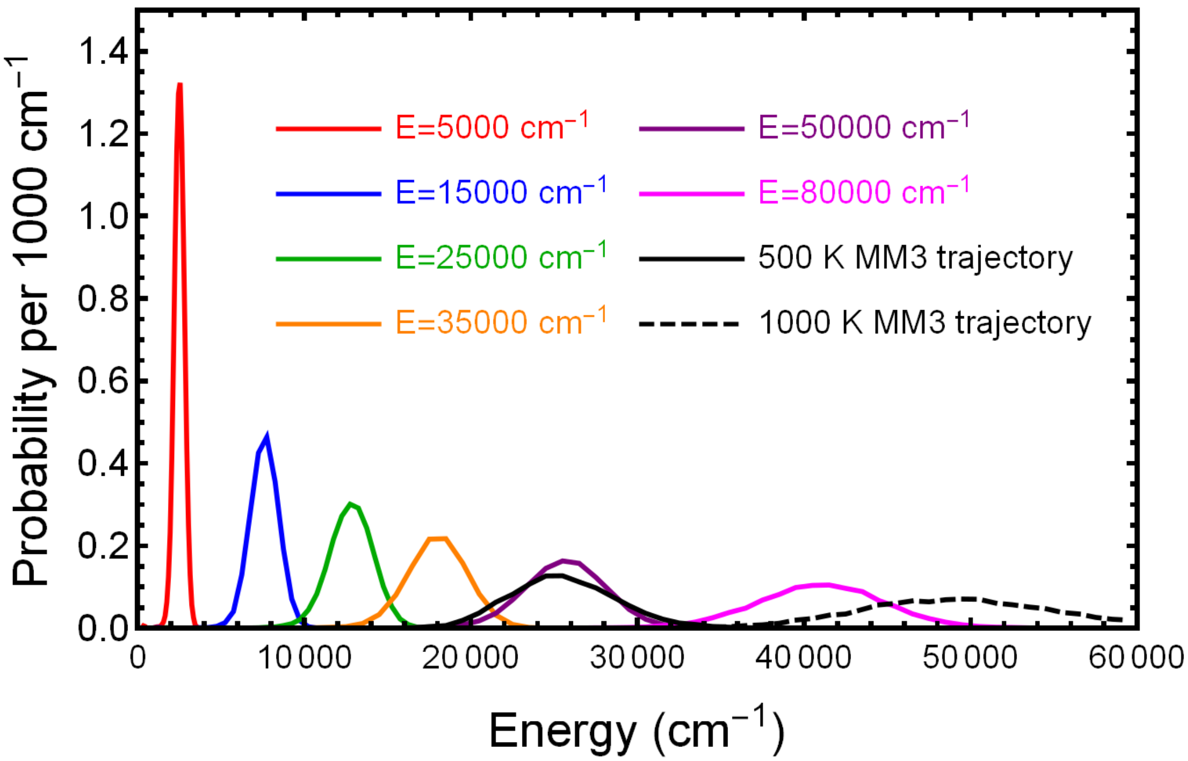

Figure SI-6: Histograms of potential energy distributions for MD trajectories run at various total energies starting from the GM. Included for reference are the histograms for two MM data sets (run at 500 K and 1000 K).

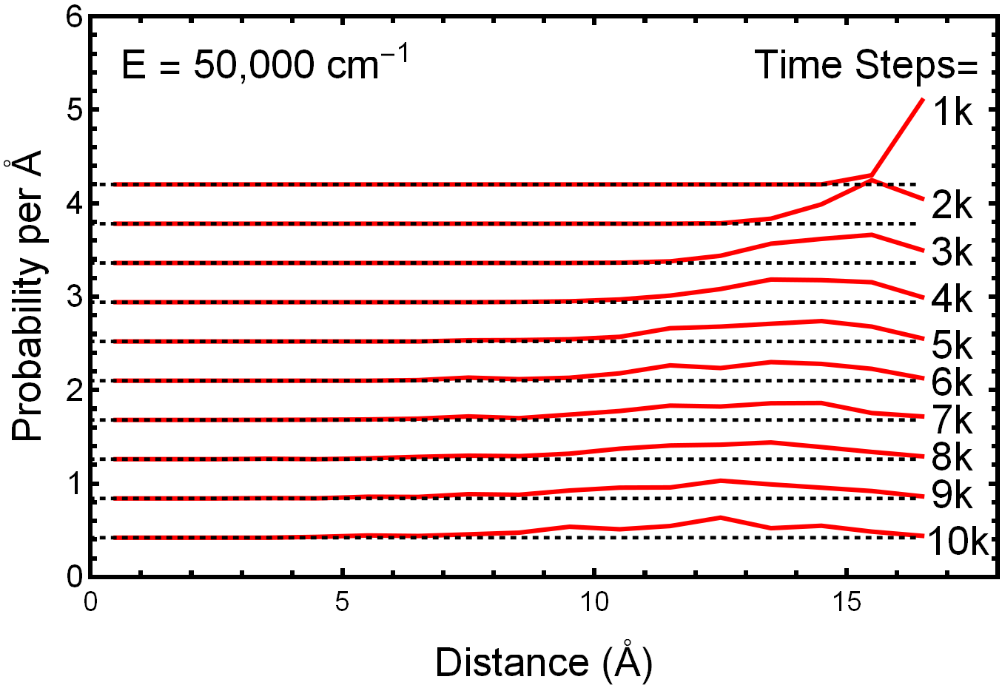

Figure SI-7: Histograms of the C1-C14 internuclear distances for MD trajectories run at a total energy of 50,000 cm^{-1} starting from the GM as a function of time steps, where each step is 5 a.u. or approximately 0.121 fs.
